# Spatiotemporal variations in cardiovascular disease mortality in China from 1991 to 2009

**DOI:** 10.1186/s12872-019-1128-x

**Published:** 2019-07-02

**Authors:** Hongyan Ren, Xia Wan, Cao Wei, Gonghuan Yang

**Affiliations:** 1 State Key Laboratory of Resources and Environmental Information System, Institute of Geographic Sciences and Natural Resources Research, Chinese Academy of Sciences, Beijing, 100101 China; 2 Institute of Basic Medical Sciences, Chinese Academy of Medical Sciences and School of Basic Medicine, Peking Union Medical College, #5 Dong Dan San Tiao, Dongcheng District, Beijing, 100005 China

**Keywords:** Coronary heart disease, Stroke, Spatiotemporal variation, Ordinary kriging, Epidemiological changes

## Abstract

**Background:**

In China, the spatiotemporal variations in cardiovascular disease (CVD) mortality are seldom characterized to understand their epidemiological features. It would be helpful to evaluate the performance of CVD-related interventions for subsequent adjustments.

**Methods:**

The 2010 Census data as well as the coronary heart disease (CHD) and stroke mortality data from the Disease Surveillance Points (DSPs) were used to calculate the age standardized death rates (ASDRs) of CVD in the DSP counties during 1991–1995, 1996–2000, 2004–2005, and 2006–2009. The ordinary kriging (OK) method was used to estimate the county-level death rates of CHD and stroke and achieved satisfactory results.

**Results:**

The goodness-of-fit between measured and estimated values of CVD mortality was significant at the 0.01 level (0.34 < R^2^ < 0.98). The counties with high CHD death rates (> 75 per 10^5^) were located in the Northwest, North, and Northeast in 1991–2000 and then extended toward the North, Central, and South, yielding an inverted-triangle-shaped area in 2004–2009. The counties with a CHD death rate increase greater than 100% were concentrated in the Northeast and South. The Northeast-Southwest regions with a high stroke death rate gradient (> 150 per 10^5^) narrowed in1991–2000, was followed by a slight expansion during 2004–2005, finally reducing in 2006–2009. The counties with a stroke mortality increase greater than 100% were scattered across the Northeast, Northwest, Central, and South.

**Conclusion:**

The epidemiological characteristics of both CHD and stroke mortality in China was spatiotemporally featured on the county level during 1991–2009.

## Background

Cardiovascular diseases (CVDs) are caused by abnormalities in the heart and blood vessels. The two most important CVDs are coronary heart disease (CHD) and stroke. It has been estimated that over the past three decades, CHD mortality has decreased by more than 50% in many developed countries such as Finland, England, the United States, Canada, Australia, New Zealand, France, and Japan [1–4]; however, the situation remains challenging in developing nations, which account for more than 60% of the global disease burden [5]. In China, CVD is the leading cause of death and disability among adults [6, 7]. In addition to aging and population growth over the coming 20 years, projected unfavorable trends in blood pressure, total cholesterol, diabetes, and body mass index may accelerate the epidemic [8]. Therefore, CVD should be actively and effectively addressed by future chronic disease prevention and control measures in China.

Many studies have revealed that the epidemiological characteristics of CVD show obvious geographical disparities, and its epidemiological pattern has changed significantly along with the rapid economic development in China [9–15]. Recent studies have focused primarily on temporal variations in epidemiological characteristics (incidence, fatality, and mortality) or its variations at the regional, provincial, or municipal levels [9, 13, 16–20]. Counties are the basic administrative unit in China. To the best of our knowledge, no previous studies have examined the spatiotemporal variations in CVD mortality across China at the county level. This lacuna makes it difficult to recognize the areas of high CVD mortality and those with great increases in CVD mortality, which in turn restricts the timely adjustment of intervention strategies for this epidemic in these areas.

The aims of this study sought to describe the spatial and temporal variations in the CHD and stroke mortality rates of all the counties in China in 1991–2009 using a geospatial tool. This study might help to understand the epidemiological characteristics of CHD and stroke across different regions as well as provide a reference for evaluating the effectiveness of CVD prevention control measures to develop future local intervention plans.

## Methods

### Data sources and pretreatment

**Fig. 1:**
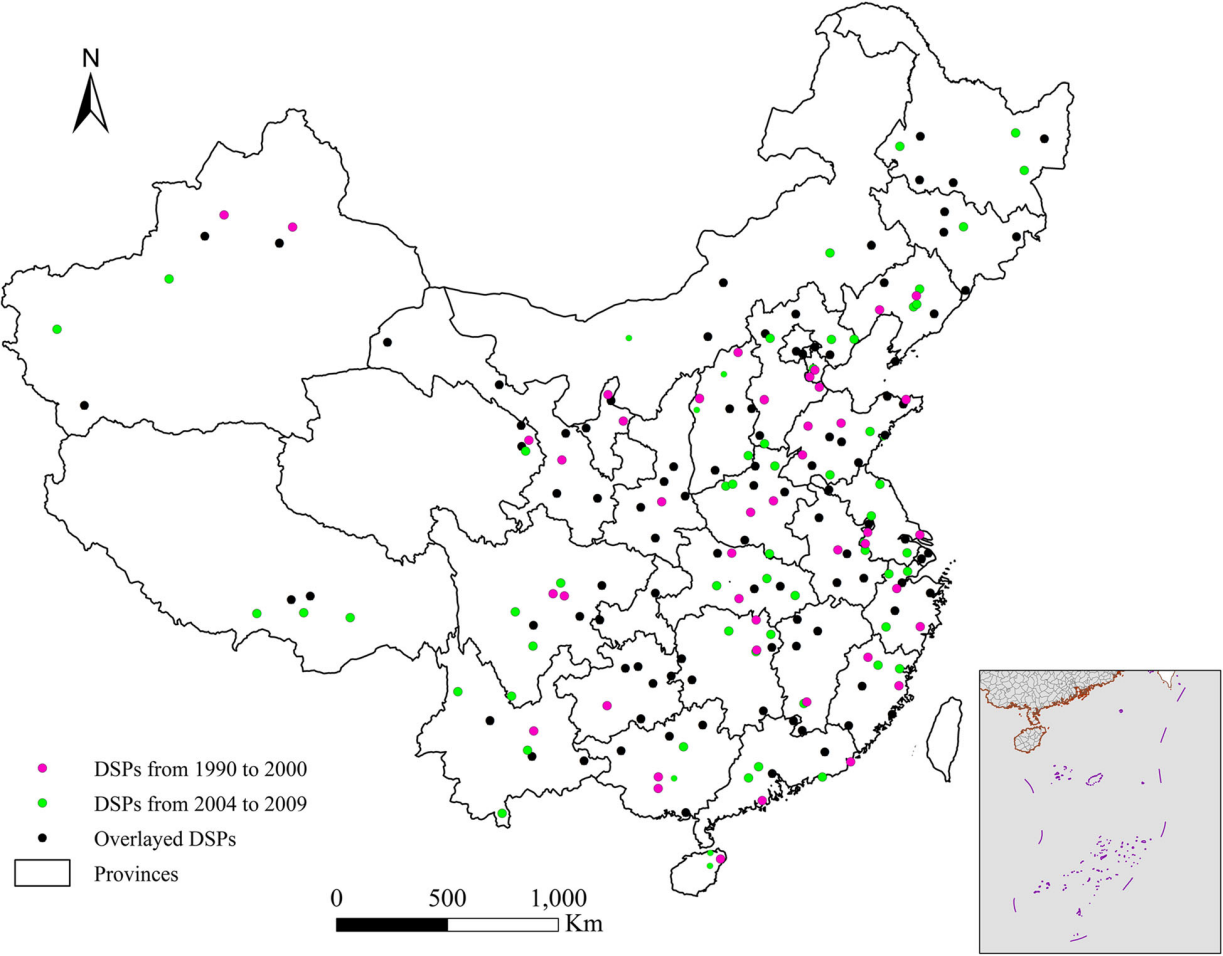
Distribution of the Death Surveillance Points (DSPs) system during 1990–2009

The mortality data in this study originated from the National Disease Surveillance Points (DSPs) system, which was established to record the causes of death in China in 1990. This system covers a population of 10 million people across 145 locations in all of the provinces using multiple-stratified random sampling [21] (Fig. 1), and this system was expanded in 2001 to cover 71.4 million people across 161 locations [21], which included 103 of the original locations and 58 new locations [22]. The new DSP system was expanded to cover the whole district of a city or county instead of one or two residential district(s) or town(s) at each location.

Recently, more people have migrated from rural to urban areas for jobs. When these people die, their death certificate is registered in their hometown because of the *Hukou* restriction. Because deaths are occasionally registered late for people who die outside of their hometowns, we analyzed the data that were recorded before 2010. Before the analysis, the data were adjusted by garbage code redistribution and underreporting rates and defined using the ICD-9 and ICD-10 international classifications of diseases. The detailed information is also described in other works [23, 24]. Every 3 years from 1991 to 2000 and 2006 to 2008, an independent survey based on “capture-mark-recapture” methods was conducted to estimate under-reporting, and mortality estimates were adjusted accordingly [21, 25]. Because the surveillance population at each point was not large prior to 2000, the average 5-year mortality rates were used for each point from 1991 to 2000. Because the system was expanded, the first 3 years of data (i.e., from 2001 to 2003) were missing. Therefore, the data from 2004 to 2005 and 2006–2009 were used after 2000 in the current study.

To verify the increasing and decreasing trends of these diseases, the age-standard death rates (ASDR) of CVD in DSP counties for each year were computed based on the Chinese population structure obtained from the 2010 census data of China (http://www.stats.gov.cn/tjsj/pcsj/rkpc/6rp/indexch.htm). A normal distribution test was performed for the county-level ASDR of CHD and stroke during four periods (1991–1995, 1996–2000, 2004–2005, and 2006–2009). The county-level ASDR data was log-transformed for years when the data were not normally distributed.

### Ordinary kriging (OK) method

According to Tobler’s First Law of Geography [26], points closer in spatial position are more likely to share similar characteristics. Spatial interpolation can be used to estimate data from non-sampling areas (based on the data from sampling areas). The OK method is a spatial interpolation method that takes the positional relationship between the data from sampling areas as well as that between unknown and sampling areas into consideration. The OK method is preferentially used because of its high accuracy and estimation stability [27, 28], especially when there was no obvious dominant trend of sampling data. Like other diseases [29, 30], the epidemiological characteristics of CVD show significant geographic disparities. Thus, the OK method was used to estimate the spatial distribution of the county-level ASDR of CVD in unknown areas nationwide.

Based on the variogram function theory and a structural analysis, the OK method is used to perform an unbiased, optimal estimation of regionalized variables within a limited area by taking the relative positional relationship between known points as well as that between known and unknown points into consideration. The estimate of a variable at point *x*, i.e., *Z*^∗^(*x*), can be obtained from the linear combination of *n* valid observed values, i.e., Z (*x*_*i*_), within the range of influence of the point:

1 $$ {Z}^{\ast }(x)=\sum \limits_{i=1}^n{\delta}_iZ\left({x}_i\right) $$

where *δ*_*i*_ is the weight assigned to the observed values Z(*x*_*i*_) (the ASDRs of CVD in DSP counties) and indicates the contribution of the observed values to the estimated value *Z*^∗^(*x*) (the ASDRs of CVD in non-DSP counties)*.* The sum of *δ*_*i*_ is 1, and the variable is calculated using a semi-variogram based on the assumption of unbiased and optimal estimation.

Spatial interpolation using the OK method was performed using ArcGIS 10.0 software (Esri,Redlands, CA, USA). The accuracy of the spatial interpolation was evaluated by leave-one-out cross validation and a root mean square error (RMSE) analysis as follows:

2 $$ \mathrm{RMSE}=\sqrt{\frac{\sum {\left({y}_m-{y}_e\right)}^2}{n}} $$

where y_m_ is the observed ASDR of CHD or stroke of DSP counties, and y_e_ is the value estimated using the OK method. We calculated the goodness-of-fit (R-square) between the estimated and measured data with regard to ASDR of CVD in the total population and gender group in DSP counties across different periods. For clarity of comparison, the RMSEs were divided by the mean ASDR of CHD or stroke and are shown as relative RMSEs. A higher R-square and a relatively lower RMSE denotes a better estimation performance.

### Index calculation

Based on the estimated values in China from 1991 to 1995 (baseline) and 2006–2009, the temporal variations in the county-level CVD death rates (ASDRs) between the first period (1991–1995) and the fourth period (2006–2009) were calculated using the following formula:

3 $$ \mathrm{Difference}\ \mathrm{of}\ \mathrm{Mortality}=\frac{\left( Average\ {Death\ Rate}_{2006-2009}- Average\ {Death\ Rate}_{1991-1995}\right)}{\left( Average\ {Death\ Rate}_{1991-1995}\right)}\times 100\% $$

## Results

**Fig. 2:**
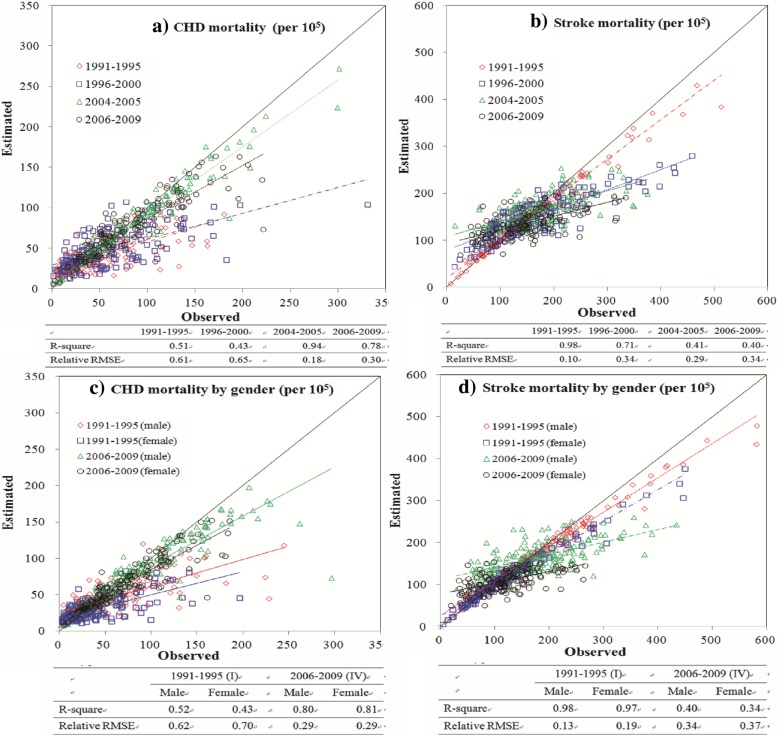
Comparasion of the estimated vs. observed death rates of coronary heart disease (CHD) and stroke by ordinary kriging (OK) method in the total population ((**a**) CHD and (**b**) stroke) ;and by gender ((**c**) CHD and (**d**) stroke) during four periods (1991 - 1995, 1996 - 2000, 2004 - 2005, and 2006 - 2009)

The spatial estimation of CVD death rates (ASDRs) at the county level in China achieved satisfactory results according to the R-square and relative RMSE values shown in Fig. 2. Moreover, because of higher R-square and lower relative RMSE values, the estimation of the CHD death rates from 1991 to 2000 was much more satisfactory than that from 2004 to 2005 and 2006–2009. The performance of the stroke estimation changed in the reverse direction during the same period. In comparison, the estimation of the CHD and stroke death rates displayed the same temporal differences by gender as they did in the total population.

**Fig. 3:**
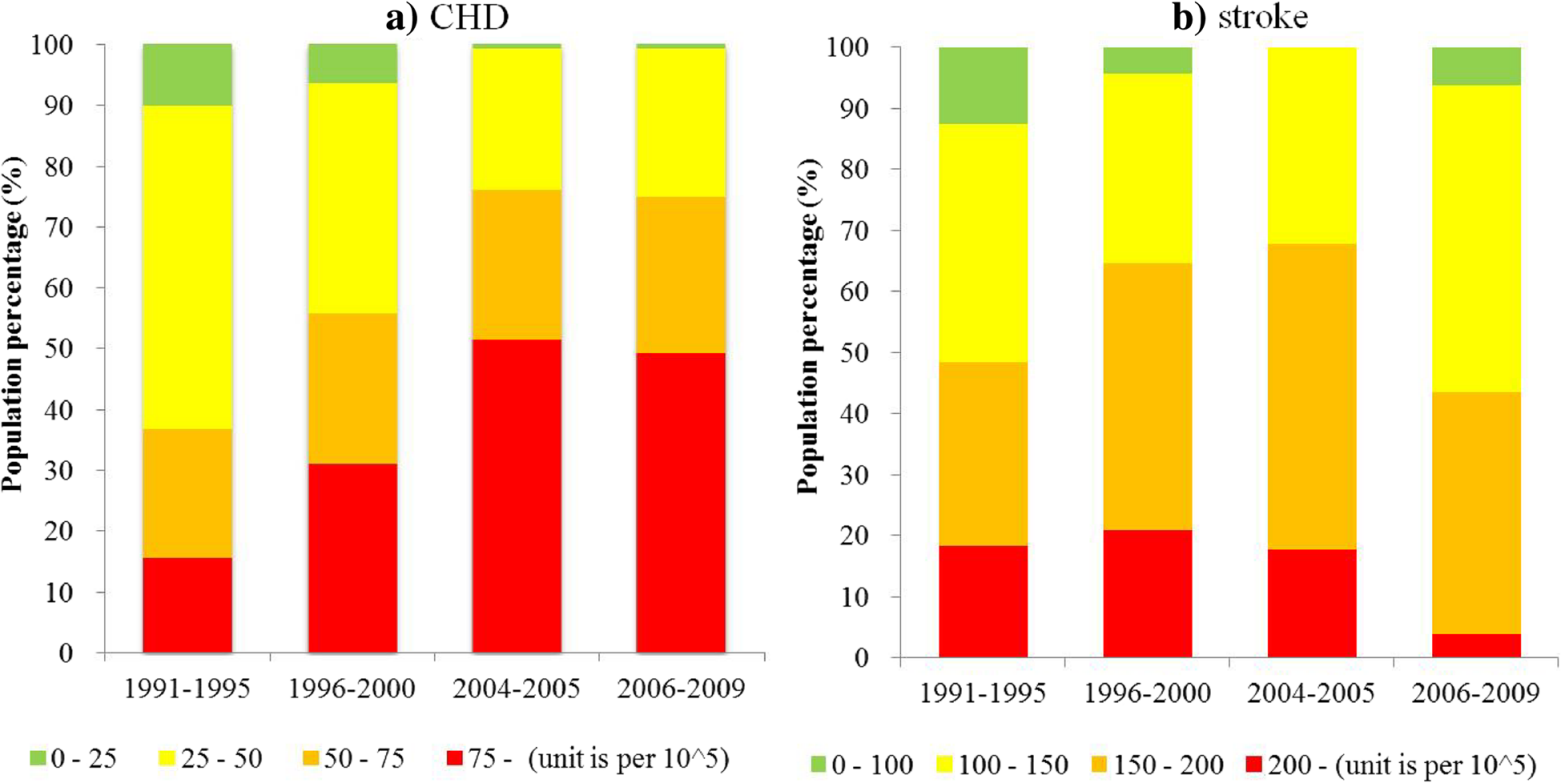
Temporal changes of the proportion of the population in the counties grouped by CVD death rates to the national population for coronary heart disease (CHD, **a**) and stroke (**b**) during the four periods (1991–1995, 1996–2000, 2004–2005, and 2006–2009)

We classified the estimated death rates of CHD into four grades: 0–25, 25–50, 50–75, and 75 – (per 10^5^). The estimated death rates of stroke were also classified into four grades: 0–100, 100–150, 150–200, and 200 – (per 10^5^). All of the counties were divided into four groups so that the populations (taken from the Provincial or Municipal Statistical Yearbook) in these grouped counties were summed across different periods. Accordingly, the proportion of the populations in these grouped counties to the national population was calculated to indicate the temporal trend of the CVD death rates in China from 1991 to 2009 (Fig. 3).

In terms of these proportions, CHD and stroke presented different temporal trends, espeically at the fourth grade (CHD: 75 per 10^5^~; stroke: 200 per 10^5^~). As Fig. 3 – a illustrated, the proportion of the population in counties with the fourth grade of CHD death rates (75 per 10^5^~) dramatically increased from 15% to approximately 50% from 1991 to 2005, whereas those of the first and second grades clearly declined. In contrast, the proportion of the population with the fourth grade of stroke death rates (above 200 per 10^5^) was retained at a level of 20% before an obvious decrease to approximately 4% from 2006 to 2009 (Fig. 3b). These results indicate that the period from 2004 to 2005 was the temporal inflection for both CHD and stroke death rates in China during 1991–2009.

**Fig. 4:**
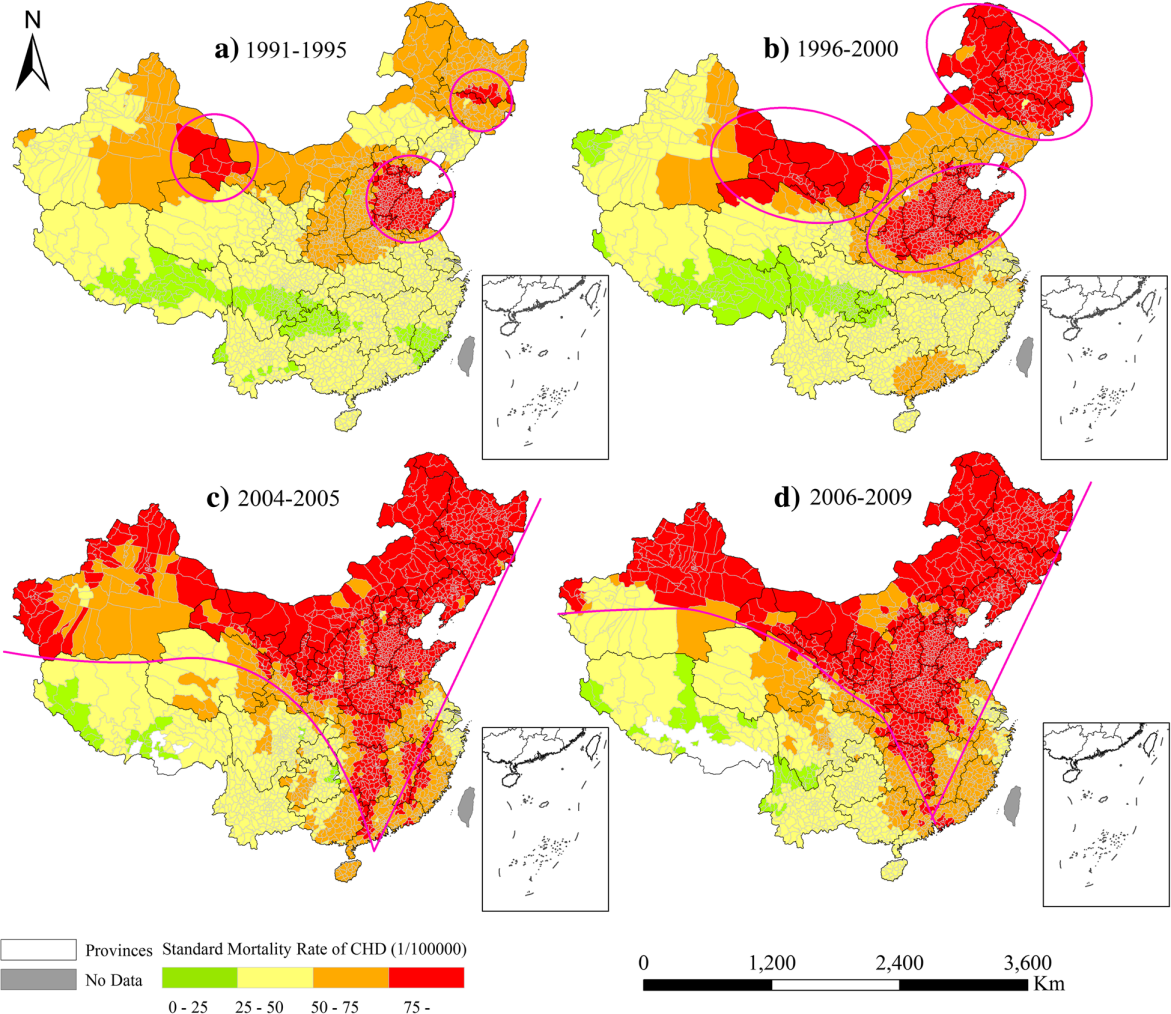
Spatial variations of estimated death rates of coronary heart disease (CHD) during 1991–1995 (**a**), 1996–2000 (**b**), 2004–2005 (**c**), and 2006–2009 (**d**)

In addition to differentiated temporal trends, the spatial patterns of the county-level death rates (estimated ASDRs of CHD and/or stroke) and their changes tended to displayed different features. During the baseline period (1991–1995), the counties with higher CHD death rates than 75 per 10^5^ were mostly distributed in three clustered areas (Fig. 4a). After this period, these counties showed an obvious spatial expansion around these three clusters greater from 1996 to 2000 (Fig. 4b). In particular, these clustered areas were then gradually jointed from 2004 to 2005 and 2006–2009, resulting in an inverted triangle region across this nation (Figs. 4c and d).

**Fig. 5:**
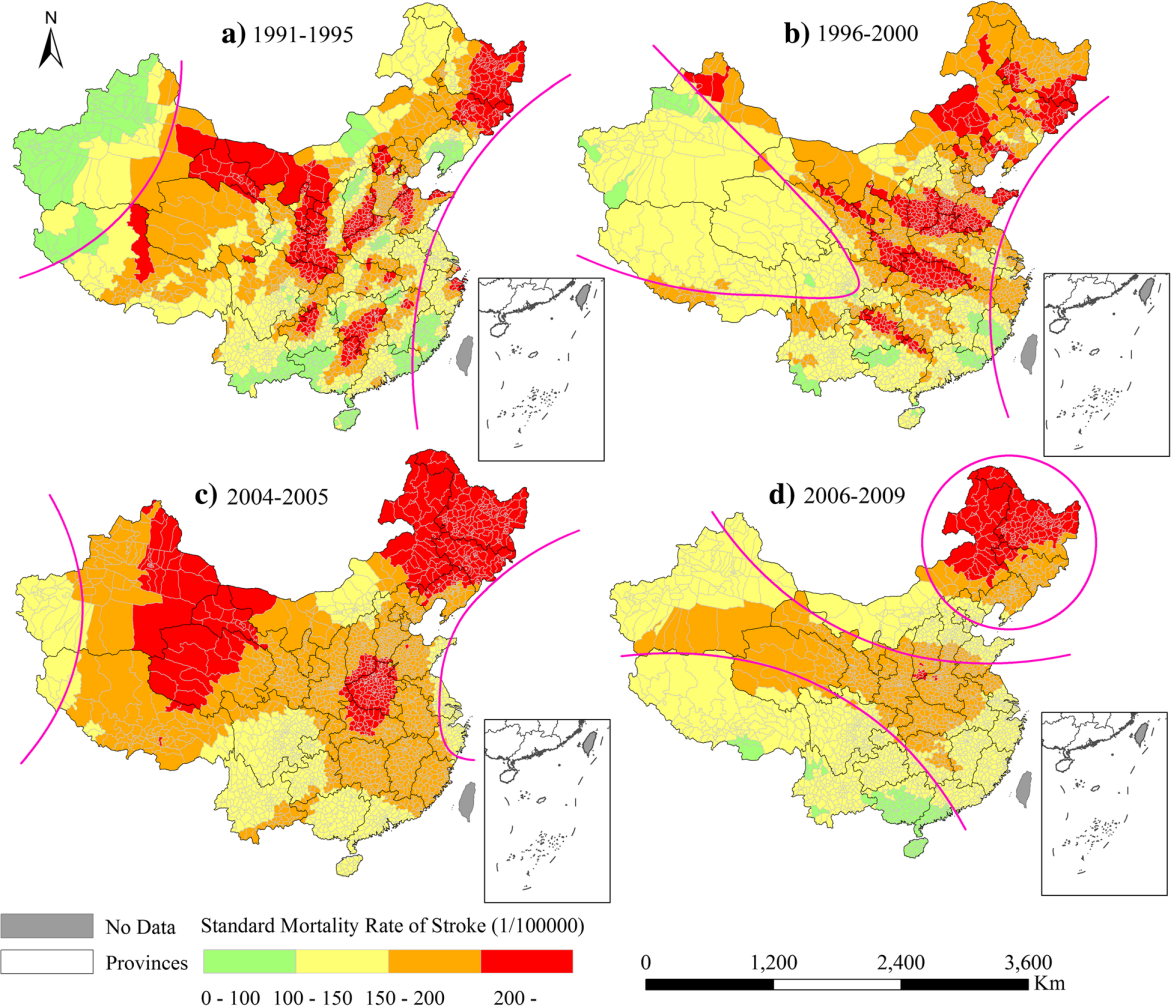
Spatial variations of estimated death rates of stroke during 1991–1995 (**a**), 1996–2000 (**b**), 2004–2005 (**c**), and 2006–2009 (**d**)

In comparison, the spatial variations of the estimated stroke death rates differed from that of the CHD at the county level. As Fig. 5a illustrated, the counties with higher stroke death rates than 150 per 10^5^spatially formed a wide belt from the Southwest to the Northeast across China from 1991 to 1995. From 1996 to 2000 (Fig. 5b), this belt narrowed in its central portion (i.e., the Qinghai, Sichuan, and Gansu provinces), became wider in the West and Southeast from 2004 to 2005 (Fig. 5c), and then decreased and split into two regions from 2006 to 2009 (Fig. 5d). One of these regions was a slim belt from the West to East. The other was the Northeast region (North Heilongjiang and Northeast Inner Mongolia) where stroke mortality was much greater than 200 per 10^5^. In addition, there were some counties in Northeast China continuously possessing higher death rates of both CHD (Fig. 4) and stroke (Fig. 5) during the four calendar periods. However, many more counties in the rest regions didn’t display a similar characterization yet.

**Fig. 6:**
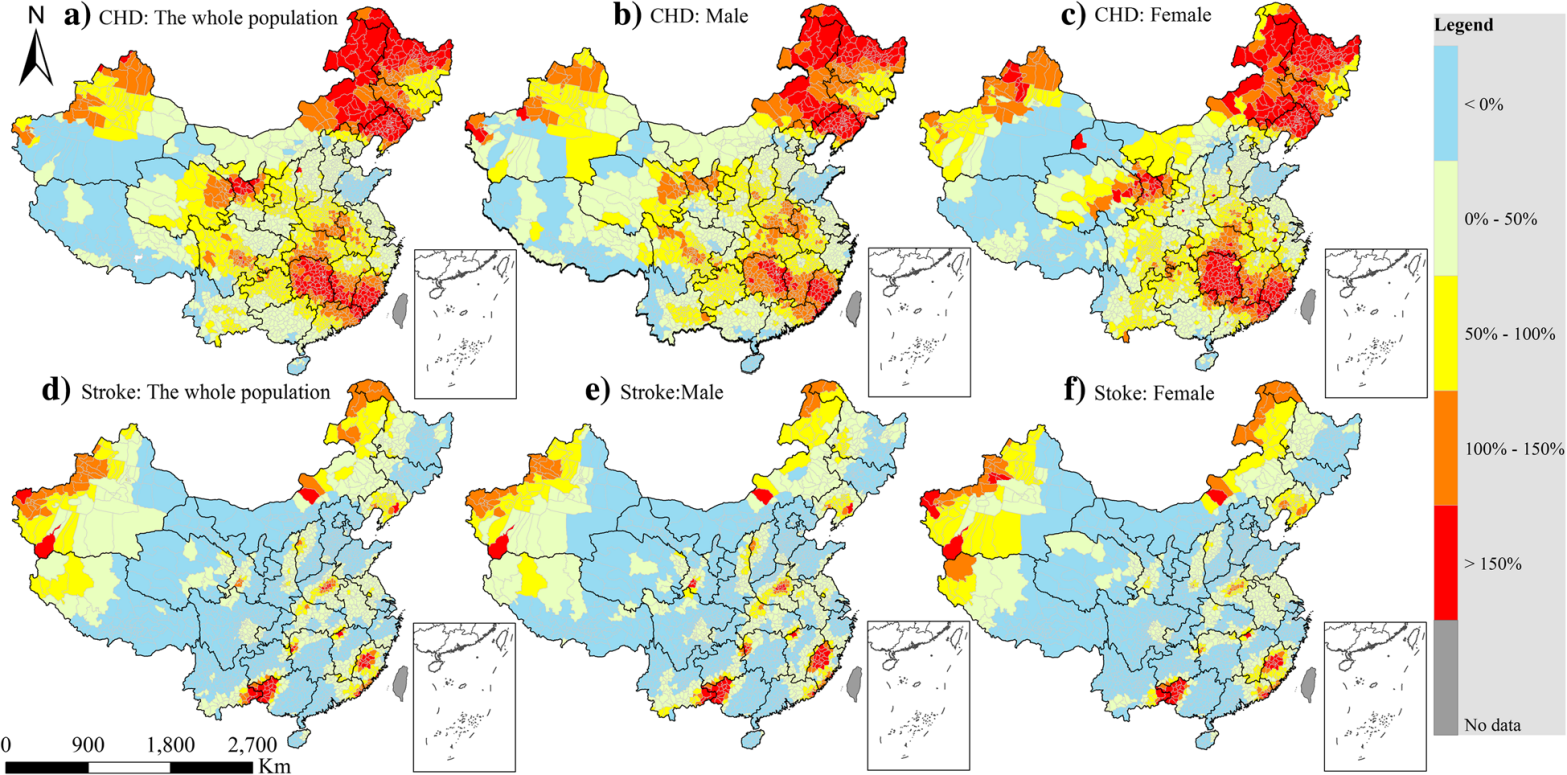
Spatial distribution of the changes of estimated death rates of coronary heart disease (CHD) and stroke among two periods (1991–1995 and 2006–2009). CHD: **a**) in the total population, **b**) male, and **c**) female; Stroke: **d**) in the total population, **e**) male, and **f**) female

According to the spatial and temporal trends of the county-level ASDRs of CVD across China, the counties with outstanding increase or decline were discriminated between the first (1991–1995) and the last periods (2006–2009). In total, there were respectively 2600 and 1300 counties with increasing death rates of CHD and stroke, approximately 91 and 44% of the total counties (Table 1). The spatial distribution of the counties that showed temporal changes of estimated CHD (Fig. 6 b and c) and stroke death rates by gender (Fig. 6 e and f) was similar to that of the total population (Fig. 6 a and d). About 800 counties showed a greater than 100% increase of CHD, mainly locating in the Northeast and the South Central regions of China (Fig. 6 a). Meanwhile, 150 counties, sparsely distributed across West Xinjiang, Northeast Inner Mongolia, Southeast Liaoning, Central Henan, West Fujian, and the junction of Yunnan-Guizhou-Guangxi (Fig. 6 d), displaying a greater of 100% increase of stroke. In contrast, about 1600 counties in the Northeast, Beijing–Tianjin–Heibei, and West regions, approximately 55% of the total counties, presented obvious decrease of stroke death rates (Fig. 6d), which made a great contribution to the reduction of the regions with high stroke death rates (Fig. 5). In addition, there were also the counties with simultaneously increasing death rates of both CHD and stroke across China (Fig. 6). However, more counties tended to present opposite trends of these two diseases.

**Table 1:** Summary of grouped counties with different changing death rates (OK-estimated) of CHD and stroke in two periods (1991–1995 and 2006–2009)

Parameters	CHD					Stroke				
	Group I	Group II	Group III	Group IV	Group V	Group I	Group II	Group III	Group IV	Group V
Number of counties	257	947	854	417	394	1597	873	249	96	54
Percentage	9%	33%	30%	14%	14%	56%	30%	9%	3%	2%
Mean mortality in 1991–1995	68.53	54.14	44.14	41.04	37.27	187.57	130.93	94.76	69.01	41.21
Mean mortality in 2006–2009	57.52	67.39	76.1	90.73	110.59	140.5	155.87	159.47	149.91	132.63
Significance (*p*-value)	< 0.001	< 0.001	< 0.001	< 0.001	< 0.001	< 0.001	< 0.001	< 0.001	< 0.001	< 0.001

According to the difference of OK-estimated death rates of CVD (CHD and stroke) in these two periods (1991–1995 and 2006–2009), all the counties were classified into five groups (I: < 0%, II: 0–50%, III: 50–100%, IV: 100–150%, and V: > 150%). Accordingly, the number of counties in each group and their percentages were respectively obtained. The paired T-test was applied for the comparison of OK-estimated CVD death rates of each group

## Discussion

This study was the first to reveal that the epidemiological characteristics of the CHD and stroke death rates in China tended to be spatially and temporally characterized at the county level. During 1991–2009, the epidemic of CHD in China became increasingly serious because of the remarkable spatial expansion of the counties with high CHD death rates, whereas that of stroke obviously declined due to the notable spatial shrinking of the counties with high stroke death rates, especially across 2004–2005. Overall, the outcome assessment of CVD-related interventions in China could benefit reasonably from the temporal and spatial clues derived from the current study. It might provide an important temporal clue for public health authorities evaluating the outcome of CVD-related interventions at different stages in China over the past decades.

Many studies have shown that the ASDR of CHD presented an outstanding ascending trend while that of stroke obviously decreased in China over the past decades [9–14, 20, 23]. It was also observed in our study. The increasingly serious status of CHD death rates was mainly attributed to the ascending prevalence of the various risk factors in China [31–34], including hypertension, smoking, dyslipidemia, diabetes, obesity, etc. Meanwhile, high rates of out-of-hospital CHD deaths (e.g., more than 70% in Beijing [35]) was another important cause of the increasing CHD status in this county. Accordingly, more and more counties/districts displayed high ASDR of CHD across China during 1991–2009. Moreover, increasingly higher ASDR of CHD was observed in some areas, especially in the Northeast and Central South regions in this study, which was also occurred in other East Asia countries [36–38].

On the contrary, the declining mortality trend for stroke in China did not match the related levels of prevalence of these similar risk factors, which may be strongly affected by the increasingly improvements of medical service system by China’s great economic development [39, 40]. In particular, the county-level hospitals in the rural regions were gradually able to carry out thrombolysis therapy and minimally invasive puncture for cerebral hemorrhage [40]. As a result, a decreasing trend of stroke death rates was observed in about 1600 counties that distributed sparsely across this country. Even so, there were still obviously ascending ASDR of stroke observed in 150 counties, which may be related to their unenlightened medical service level in some undeveloped areas (e.g., the boundary between Xinjiang and Tibet, the western part of Guangxi and Fujian).

In addition, a province-level characterization outlined a surrounding belt of nine provinces with high stroke death rates in Northern China despite that its basic data did not cover the entire country [14]. Many more studies have suggested that stroke mortality tends to show northeast–southwest gradient variations [12, 13, 41]. Interestingly, all of the regions with high stroke death rates previously reported (except Tibet) were covered by those described in our study in the 1990s, which indicates that the high stroke mortality belt in China is constantly changing. Compared with the regional, provincial, and municipal variations of CVD death rates characterized in the previous investigations [18, 20, 23, 42–48], the specific regions with higher, lower, increasing, or decreasing CVD death rates were clearly revealed at the county level in China over the past decades. These results indicated that the geographic distribution of the death rates of CVD (CHD and stroke) can be well characterized on a more detailed scale.

Certain limitations of this study warrant mentioning. First, spatial interpolation using the OK method tends to increase the lowest values and decrease the highest values, especially in regions with few and sparse surveillance points. This disadvantage might be reduced through the densification of surveillance points and an improvement of the DSP system. Second, the data obtained from a retrospective survey of the causes of death from 2004 to 2005 might be somewhat higher than the DSP data and lead to an overestimation of the causes of death such as the large area of high stroke mortality in West China during this period, which should be confirmed by future investigations. Although we have formed an overall understanding on the spatial and temporal changes of CVD mortality in China over the past two decades, certain risk factors, including blood pressure, total cholesterol, diabetes, overweight, climate change, and air pollution, should be obtained in the next step and integrated into some spatiotemporal models for further exploring the potential causes of the spatiotemporal variations in the county-level CVD mortality. However, some important clues regarding the assessment of the present CVD-related interventions were uncovered in this study.

## Conclusions

In summary, the epidemiology of CHD and stroke mortality from 1991 to 2009 in China was spatially and temporally featured at the county level. The study was the first to present critical spatial and temporal clues regarding the assessment and adjustment of CVD-related interventions in this country, and it provides useful support for exploring the spatial and temporal variations of the CVD risk factors in China.

## Data Availability

The data that support the findings of this study are available from the Chinese Center for Disease Control and Prevention (CCDC) and the Chinese Academy of Medical Sciences (CAMS). The dataset from 1991 to 2000 used and analyzed during this study from CAMS are available from the corresponding author on reasonable request. The data after 2000 that support the findings of this study are available from CCDC but restrictions apply to the availability of these data, which were used under license for the current study, and so are not publicly available. Data are however available from the authors upon reasonable request and with permission of CCDC.

## Abbreviations

ASDR: Age standardized death rate
CHD: Coronary heart disease
CVD: Cardiovascular disease
DSP: Disease surveillance points
OK: Ordinary kriging
RMSE: Root mean square error

## References

[1] Labarthe DR. Epidemiology and prevention of cardiovascular diseases: a global challenge. Sudbury: Jones and Barrlett Publishers; 1998.

[2] Cooper R, Cutler J, Desvigne-Nickens P, Fortmann SP, Friedman L, Havlik R, Hogelin G, Marler J, McGovern P, Morosco G (2000). Trends and disparities in coronary heart disease, stroke, and other cardiovascular diseases in the United States: findings of the national conference on cardiovascular disease prevention. Circulation.

[3] Reddy KS, Yusuf S (1998). Emerging epidemic of cardiovascular disease in developing countries. Circulation.

[4] Ohira T, Iso H (2013). Cardiovascular disease epidemiology in Asia: an overview. Circ J.

[5] Mackay J, Mensah G. The atlas of heart disease and stroke. In*.*: World Health Organization; 2004. https://www.who.int/cardiovascular_diseases/resources/atlas/en/.

[6] Hu SS, Kong LZ, Gao RL, Zhu ML, Wang W, Wang YJ, Wu ZS, Chen WW, Liu MB (2012). Outline of the report on cardiovascular disease in China, 2010. Biomed Environ Sci.

[7] National Center for Cardiovascular Diseases: Report on Cardiovascular Diseases in China, 2008-2009. Beijing: Encyclopedia of China Publishing House; 2010. https://www.amazon.cn/dp/B007MAKBLE.

[8] Moran A, Gu D, Zhao D, Coxson P, Wang YC, Chen CS, Liu J, Cheng J, Bibbins-Domingo K, Shen YM (2010). Future cardiovascular disease in China: markov model and risk factor scenario projections from the coronary heart disease policy model-China. Circulation Cardiovascular quality and outcomes.

[9] Truelsen T, Mahonen M, Tolonen H, Asplund K, Bonita R, Vanuzzo D (2003). Trends in stroke and coronary heart disease in the WHO MONICA project. Stroke.

[10] Jiang GH, Wang DZ, Li W, Pan Y, Zheng WL, Zhang H, Sun YV (2012). Coronary heart disease mortality in China: age, gender, and urban-rural gaps during epidemilogocial transition. Rev Panam Salud Publica.

[11] Wong Irene O. L., Cowling Benjamin J., Leung Gabriel M., Schooling C. Mary (2013). Age-Period-Cohort Projections of Ischaemic Heart Disease Mortality by Socio-Economic Position in a Rapidly Transitioning Chinese Population. PLoS ONE.

[12] Liu M, Wu B, Wang WZ, Lee LM, Zhang SH, Kong LZ (2007). Stroke in China: epidemiology, prevention, and management strategies. The Lancet Neurology.

[13] Zhao D, Liu J, Wang W, Zeng Z, Cheng J, Liu J, Sun J, Wu Z (2008). Epidemiological transition of stroke in China: twenty-one-year observational study from the Sino-MONICA-Beijing project. Stroke.

[14] Xu G, Ma M, Liu X, Hankey GJ (2013). Is there a stroke belt in China and why?. Stroke.

[15] Murakoshi N, Aonuma K (2013). Epidemiology of arrhythmias and sudden cardiac death in Asia. Circ J.

[16] Sun XG, Wang YL, Zhang N, Wang T, Liu YH, Jin X, Li LJ, Feng J (2014). Incidence and trends of stroke and its subtypes in Changsha, China from 2005 to 2011. Journal of clinical neuroscience : official journal of the Neurosurgical Society of Australasia.

[17] Jiang B, Wang WZ, Chen H, Hong Z, Yang QD, Wu SP, Du XL, Bao QJ (2006). Incidence and trends of stroke and its subtypes in China: results from three large cities. Stroke.

[18] Zhang XF, Hu DY, Ding RJ, Wang RC, Yan LX (2012). Prevalence and epidemic tendency of cardiovascular and cerebravascular disease mortality in China. Chin J Cardiol.

[19] Zhao D (1993). The epidemiology of coronary heart disease in 16 provinces of China. Multi-province cooperative Group of Cardiovascular Disease Surveillance (MONICA project). Chin J Epidemiol.

[20] Zhou M, Wang H, Zhu J, Chen W, Wang L, Liu S, Li Y, Wang L, Liu Y, Yin P, et al. Cause-specific mortality for 240 causes in China during 1990–2013: a systematic subnational analysis for the global burden of disease study 2013. Lancet. 2015. 10.1016/S0140-6736(15)00551-6 26510778

[21] Yang G, Hu J, Rao KQ, Ma J, Rao C, Lopez AD (2005). Mortality registration and surveillance in China: history, current situation and challenges. Popul Health Metrics.

[22] Zhou MG, Jiang Y, Huang ZJ (2010). Adjustment and representativeness evaluation of national disease surveillance points system. Disease Surveillance.

[23] Yang G, Kong L, Zhao W, Wan X, Zhai Y, Chen LC, Koplan JP (2008). Emergence of chronic non-communicable diseases in China. Lancet.

[24] Wan X, Ren H, Ma E, Yang G (2017). Mortality trends for ischemic heart disease in China: an analysis of 102 continuous disease surveillance points from 1991 to 2009. BMC Public Health.

[25] Wang L, Wang LJ, Cai Y, Ma LM, Zhou MG (2011). Analysis of under-reporting of mortality surveillance from 2006 to 2008 in China. Zhonghua Yu Fang Yi Xue Za Zhi (in Chinese).

[26] Tobler WR: A computer movie simulating urban growth in the Detroit region. Econ Geogr 1970, 46(ArticleType: research-article / Issue Title: Supplement: Proceedings. International Geographical Union. Commission on Quantitative Methods / Full publication date: Jun., 1970 / Copyright © 1970 Clark University):234–240.

[27] Berke O (2004). Exploratory disease mapping: kriging the spatial risk function from regional count data. Int J Health Geogr.

[28] Wu JL, Zheng XY (2007). A simulation on the emerging of birth defects in China with kriging interpolation method. Chin J Epidemiol.

[29] Li JT, Huang F, Chen W, Cheng SM (2012). The application of spatial interpolation method in the epidemiological studies. Chin J Antiuberc.

[30] Ali M, Park JK, Thiem VD, Canh DG, Emch M, Clemens JD (2005). Neighborhood size and local geographic variation of health and social determinants. Int J Health Geogr.

[31] Wang W, Zhu ML, Wang YJ, Wu ZS, Gao RL, Kong LZ, Hu SS (2013). Summary Report on Cardiovascular Diseases in China (2012). Chin Circ J.

[32] Tian ZX, Li SS, Zhang JL, Jaakkola JJK, Guo YM (2012). Ambient temperature and coronary heart disease mortality in Beijing, China: a time series study. Environ Health-Glob.

[33] Huxley RR, Hirakawa Y, Hussain MA, Aekplakorn W, Wang X, Peters SAE, Mamun A, Woodward M (2015). Age- and sex-specific burden of cardiovascular disease attributable to 5 major and modifiable risk factors in 10 Asian countries of the Western Pacific region. Circ J.

[34] Critchley J, Liu J, Zhao D, Wei W, Capewell S (2004). Explaining the increase in coronary heart disease mortality in Beijing between 1984 and 1999. Circulation.

[35] Gao YL, Su JT, Su JT, Wei ZH, Liu JL, Wang J (2012). Characteristics of out-of-hospital acute coronary heart disease deaths of Beijing permanent residents at the age of 25 or more from 2007 to 2009. Chin J Cardiol.

[36] Wong MC, Zhang de X, Wang HH (2015). Rapid emergence of atherosclerosis in Asia: a systematic review of coronary atherosclerotic heart disease epidemiology and implications for prevention and control strategies. Curr Opin Lipidol.

[37] Hata J, Kiyohara Y (2013). Epidemiology of stroke and coronary artery disease in Asia. Circ J.

[38] Yatsuya H, Li Y, Hilawe EH, Ota A, Wang C, Chiang C, Zhang Y, Uemura M, Osako A, Ozaki Y (2014). Global trend in overweight and obesity and its association with cardiovascular disease incidence. Circ J.

[39] Wang Z, Hu S, Sang S, Luo L, Yu C (2017). Age-Period-Cohort Analysis of Stroke Mortality in China: Data from the Global Burden of Disease Study 2013. Stroke.

[40] Wang W, Wang D, Liu H, Sun H, Jiang B, Ru X, Sun D, Chen Z, Wang Y (2017). Trend of declining stroke mortality in China: reasons and analysis. Stroke Vasc Neurol. vol. 2.

[41] He J, Klag MJ, Wu Z, Whelton PK (1995). Stroke in the People's Republic of China. I. Geographic variations in incidence and risk factors. Stroke.

[42] Wan X, Ren H, Yang G (2015). Mortality trends for ischaemic heart disease and stroke in China: an analysis of 102 continuous disease surveillance points from 1991 to 2009. Lancet.

[43] Liu MB, Wang W, Zhou MG (2013). Trend analysis on the mortality of cardiovascular diseases from 2004 to 2010 in China. Chin J Epidemiol.

[44] Wu ZS, Yao CG, Zhao D, Wu GX, Wang W, Liu J, Zeng ZC, Wu YK (2001). Sino-MONICA project - a collaborative study on trends and determinants in cardiovascular diseases in China, part I: morbidity and mortality monitoring. Circulation.

[45] Sun JY, Liu J, Xie XQ, Wei ZH, Wang W, Wang M, Qi Y, Liu J, Guo MN, Zhang XY (2012). Surveillance on the incidence of acute coronary events in the permanent residents of Beijing aged 25 years and more from 2007 to 2009. Chin J Cardiol.

[46] Ning XJ, Wang JH, Li ZZ (1995). Epidemiology of stroke in urban and rural areas, Tianjin, China: a six-year prospective research. Chin J Epidemiol.

[47] Huang J, Wang J, Yu W (2014). The lag effects and vulnerabilities of temperature effects on cardiovascular disease mortality in a subtropical climate zone in China. Int J Environ Res Public Health.

[48] Chen M, He S, Yan Q, Xu X, Wu W, Ge S, Zhang S, Chen M, Xia N (2017). Severe hand, foot and mouth disease associated with Coxsackievirus A10 infections in Xiamen, China in 2015. J Clin Virol.

